# High‐dose IgG suppresses local inflammation and facilitates functional recovery after olfactory system injury

**DOI:** 10.1002/acn3.51554

**Published:** 2022-05-19

**Authors:** Kohei Nishida, Masayoshi Kobayashi, Eisuke Ishigami, Kazuhiko Takeuchi

**Affiliations:** ^1^ Department of Otorhinolaryngology‐Head and Neck Surgery Mie University Graduate School of Medicine Tsu Japan

## Abstract

**Objective:**

Head trauma can be a cause of refractory olfactory dysfunction due to olfactory nervous system injury. Anti‐inflammatory treatment using steroids or anti‐cytokine agents is known to contribute to functional recovery of the central and peripheral nervous systems in injury models, while there is a concern that they can induce adverse reactions. The present study examines if high‐dose immunoglobulin G (IgG) can facilitate olfactory functional recovery following injury.

**Methods:**

Olfactory nerve transection (NTx) was performed in OMP‐tau‐lacZ mice to establish injury models. High‐dose IgG was intraperitoneally injected immediately after the NTx and histological assessment of recovery within the olfactory bulb was performed at 5, 14, 42, and 100 days after the drug injection. X‐gal staining labeled degenerating and regenerating olfactory nerve fibers and immunohistochemical staining detected the presence of reactive astrocytes and macrophages/microglia. Olfactory function was assessed using an olfactory avoidance behavioral test.

**Results:**

High‐dose IgG‐injected mice showed significantly smaller areas of injury‐associated tissue, fewer astrocytes and macrophages/microglia, and an increase in regenerating nerve fibers. An olfactory avoidance behavioral test showed improved functional recovery in the IgG‐injected mice.

**Interpretation:**

These findings suggest that high‐dose IgG could provide a new therapeutic strategy for the treatment of olfactory dysfunction following head injuries.

## Introduction

Olfactory dysfunction lowers our quality of life and can be life‐threatening because of the inability to detect hazardous events such as fire, gas leak, and spoiled food intake.^1^, ^2^ Head trauma is one of the major causes of olfactory dysfunction due to overextension, distortion, and tearing of the olfactory nerves and contusions of the olfactory bulbs and orbitofrontal regions of the brain.^3^ A major problem with traumatic olfactory dysfunction is the poor prognosis for recovery. Although the olfactory system has a remarkable capacity for neural regeneration and recovery after injury, the clinical improvement rate for olfactory dysfunction in patients with head trauma is only 10–38%^4^, ^5^, ^6^, ^7^, ^8^ while that with chronic rhinosinusitis and allergic rhinitis is reported to be 68–86%.^9^, ^10^, ^11^ Therefore, development of therapeutic management for olfactory dysfunction is an important clinical issue.

We previously reported using an olfactory nerve injury model in mice that anti‐inflammatory treatment with steroids, cytokine antagonists as anti‐interleukin‐6 (IL‐6) receptor antibody and tumor necrosis factor (TNF)‐α blocker, and anti‐high mobility group box 1 (HMGB1) antibody during the acute phase of injury is effective in suppressing the inflammatory reaction and local glial scar formation and improves recovery outcomes after olfactory nerve transection (NTx).^12^, ^13^, ^14^, ^15^ In clinical practice, however, these drugs are not typically used for the treatment of head injury patients. Several studies reported that steroids do not have a significant efficacy on morbidity and mortality in patients with severe head injury and there are concerns that steroids may cause serious side effects such as hypertension, hyperglycemia, infection, bone necrosis, and psychosis.^16^, ^17^, ^18^ Although there are fewer concerns about anti‐IL‐6 receptor antibody and TNF‐α blocker use, their administration may sometimes induce severe infection due to excessive suppression of the immune system.^19^, ^20^ In addition, anti‐HMGB1 antibody agents for human administration have not yet been developed.

Immunoglobulin G (IgG) is known to exhibit many immune‐modulatory properties and is clinically used for the treatment of many neurological diseases such as Guillain–Barre syndrome, chronic inflammatory demyelinating polyneuropathy, and multiple sclerosis.^21^ Recent studies reported that high‐dose IgG plays a neuroprotective role by reducing pro‐inflammatory cytokines and chemokines and contributes to functional and structural recovery of the central nervous system in animal models of traumatic brain and spinal cord injury.^22^, ^23^, ^24^

The present study was designed to investigate if therapeutic intervention using high‐dose IgG is effective in improving recovery outcomes in the olfactory system following injury in mice. We used histological techniques to examine the efficacy of high‐dose IgG on recovery outcome by measuring the degree of degeneration and regeneration of olfactory nerve fibers and the amount of injury‐associated tissue (glial scar), reactive astrocytes, and macrophages/microglia. We also administered an olfactory function test using avoidance conditioning behavior to odorants in order to determine if structural recovery parallels functional recovery in the olfactory system following therapeutic intervention.

## Methods

### Experimental animals

This study was performed using a transgenic strain of mice (OMP‐tau‐lacZ mice) obtained from the Jackson Laboratory (Bar Harbor, ME, USA). This strain is derived from C57BL/6 mice and the gene sequence encoding the olfactory marker protein (OMP) has been replaced with a tau‐lacZ reporter gene.^25^ The OMP is expressed in all mature olfactory neurons^26^ and the replacement with tau‐lacZ reporter gene enables the visualization of olfactory nerve fibers and their projections to olfactory bulb glomeruli. The advantage of using these mice is that a histological assessment of degenerating and regenerating olfactory nerve fibers can be performed using a standard method for staining and light microscopy. Although the functional significance of OMP is not fully understood, previous studies have shown that OMP‐tau‐lacZ mice are capable of recovering olfactory function after olfactory nerve injury.^13^, ^14^, ^15^

### Surgical procedure

**Figure 1:**
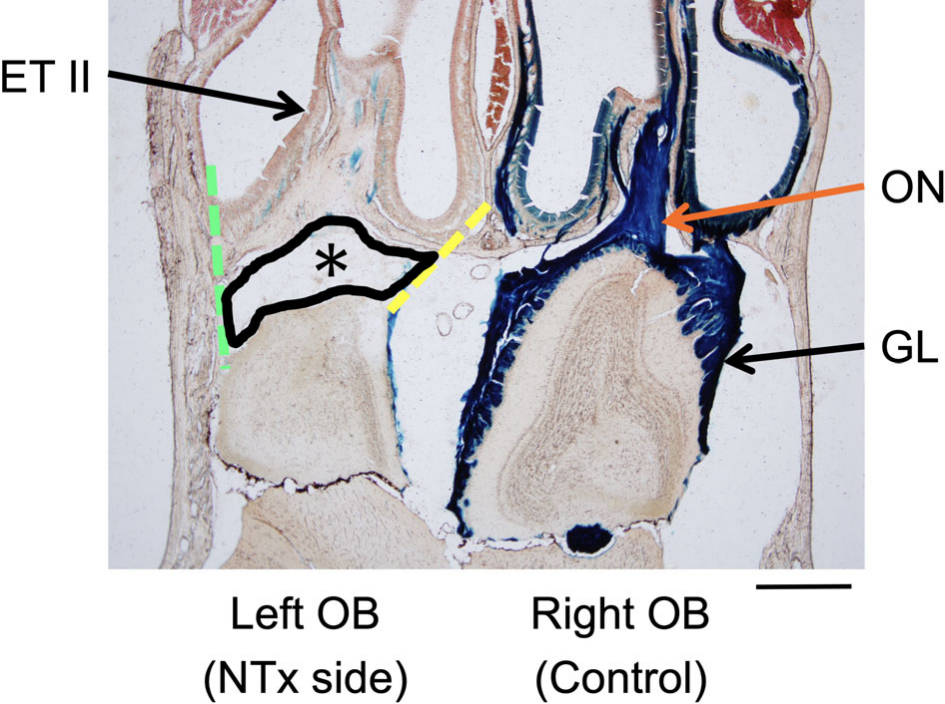
An experimental mouse model of severe olfactory bulb deafferentation injury. A horizontal section through the nasal cavities and olfactory bulbs illustrating differences observed between the lesioned (left) and control (right) sides at 5 days after a nerve transection (NTx) injury. The area of injury‐associated tissue (enclosed by a black line, *) was measured and quantified within an area surrounded by the cribriform plate (an anterior margin), anterior edge of the olfactory bulb (a posterior margin), a line connecting posterior end of the nasal septal mucosa and the anteromedial corner of the olfactory bulb (medial margin, yellow dotted line), and a line connecting posterolateral end of the most lateral sinus and the anterolateral corner of the olfactory bulb (lateral margin, green dotted line), using ImageJ software (ver. 1.53a, NIH, USA). The olfactory nerves and their projections to glomeruli are labeled using an X‐gal staining method (blue color**)**. GL, glomerular layer; OB, olfactory bulb; ON, olfactory nerve; ET II, endoturbinate II. Calibration bar = 500 μm.

Both male and female adult mice were used in this study and randomly assigned to experimental groups. Mice were anesthetized with sodium pentobarbital (80 mg/kg, *ip*). Under sufficient anesthesia, a frontal craniotomy was performed to expose the olfactory bulbs. An olfactory nerve transection procedure (NTx) was performed between the olfactory bulb and cribriform plate using a curved rigid stainless steel blade to generate a severe olfactory nerve injury model.^12^ For histological assessments, the NTx procedure was performed only on the left side (injury side) of the animal while the right side (right olfactory bulb and nerves) remained intact and served as an internal histological control (Fig. 1). For olfactory function assessment, a bilateral NTx was performed, cutting the olfactory nerves to the right and left olfactory bulbs, resulting in a complete loss of smell (anosmia). After the NTx procedure was complete, the skin incision was sutured and the animal was closely monitored until it was awake and fully recovered from anesthesia. All protocols and surgical procedures for this study were reviewed and approved by the Institutional Animal Care and Use Committee of Mie University.

### IgG injection

To investigate the associations among IgG, the inflammatory reaction, and nerve regeneration after injury, the rat IgG solution (11.4 mg/ml, Jackson ImmunoResearch, USA) was injected intraperitoneally just after the NTx. It was purified from the serum of nonimmunized animals and polyclonal IgG. To determine if there is a dose‐dependent effect of the drug, low (200 mg/kg) and high (400 mg/kg) doses of the IgG were used, making them comparable to a dose used in a previous study.^22^, ^23^ The dose of IgG used in this study was set equal to that used in clinical practice in human.^21^ For control animals, the vehicle (35 ml/kg of saline) was injected intraperitoneally. We collected data from six mice for each of the three treatment groups and each of four recovery time points (Day 5, 14, 42, and 100) for a total of 72 mice (6 mice × 3 treatments × 4 recovery time points).

### Tissue preparation

For histological assays, on the assigned post recovery day mice were anesthetized with sodium pentobarbital (80 mg/kg, *ip*) and fixed by intracardiac perfusion using 4% paraformaldehyde in phosphate buffer after a saline rinse. The nasal cavity and anterior portion of the skull were removed *en bloc* and postfixed by immersion in 4% paraformaldehyde for 45 min and then placed in 0.5 M EDTA (ethylenediaminetetraacetic acid) for decalcification for 14 days. The tissue was cryoprotected with 30% sucrose for 2 days, then immersed in embedding compound, quickly frozen in a −80°C freezer, and sectioned on a cryostat. Serial horizontal sections through the nasal cavities and olfactory bulbs along dorsum nasi were cut at 30 μm and mounted on glass slides.

### X‐gal staining

Tissue sections were washed at room temperature with buffer A [100 mM phosphate buffer (pH 7.4), 2 mM MgCl_2,_ and 5 mM EGTA (ethylene glycol tetraacetic acid)] once for 5 min and then a second time for 25 min. This was followed by two 5 min washes with buffer B [100 mM phosphate buffer (pH 7.4), 2 mM MgCl_2_, 0.01% sodium deoxycholate, and 0.02% Nonidet P40]. The blue X‐gal reaction was generated overnight in the dark by exposure to buffer C (buffer B, with 5 mM potassium ferricyanide, 5 mM potassium ferrocyanide, and 1 mg/ml of X‐gal). The X‐gal reaction was stopped by two 5 min washes in phosphate buffer.

### Measurement of injury‐associated tissue and nerve recovery

After confirming the appearance of the blue X‐gal reaction, tissue sections were counterstained with a 1% Neutral Red solution. Sections were examined and digitized using charge‐coupled device (CCD) photomicroscopy. Areas of injury‐associated tissue, including inflammatory cells and glial scar tissue, were identified along with blue (X‐gal) labeled olfactory nerve endings within the glomerular layer of the olfactory bulb (Fig. 1). The area of injury‐associated tissue was outlined on digital images of tissue sections and quantified using ImageJ (ver. 1.53a, National Institute of Health [NIH], USA) software. For the measurement of injury‐associated tissue, we targeted an area that was surrounded by the following four margins: the cribriform plate as an anterior margin, anterior edge of the olfactory bulb as a posterior margin, a line connecting posterior end of the nasal septal mucosa and the anteromedial corner of the olfactory bulb as a medial margin, and a line connecting posterolateral end of the most lateral sinus and the anterolateral corner of the olfactory bulb as a lateral margin. Since this targeted area is where the olfactory nerve fibers normally run from the sinonasal mucosa to the olfactory bulb before NTx, as seen in the control side, the tissue levels in this area can be associated with the degree of nerve degeneration and regeneration.

The area (mm^2^) of tissue observed between the cribriform plate and olfactory bulb (Fig. 1) was measured in two representative horizontal sections (sections A and B) from each animal and averaged. Section A was selected to represent the dorsal level. At this particular level, a large olfactory nerve bundle is observed passing from endoturbinate II through the cribriform plate to the olfactory bulb (Fig. 1). Section B represented a more ventral level. At this level, endoturbinate III attaches to the cribriform plate. The area measurements from NTx mice at each of the four recovery time points were used to compare mean values for injury‐associated tissue. The levels of olfactory nerve degeneration and regeneration were assessed by comparing changes in the amount of blue X‐gal staining in the glomerular layer on the left (NTx injury side) to that on the right (control) side. Horizontal olfactory bulb sections (Sections A and B) were also used to obtain measurements of^1^: the glomerular layer perimeter distance (G‐P distance), a continuous line passing through the center of all the glomeruli within the bulb section, and^2^ the total length of glomerular segments along the perimeter that were labeled with the blue X‐gal stain (G‐X‐gal distance). The ratio of the X‐gal‐stained distance (G‐X‐gal distance) to the total perimeter of the glomerular layer (G‐P distance) was obtained for both the NTx injury and control sides. Changes in the blue X‐gal nerve staining on the NTx injury‐left side were expressed as percentage of the X‐gal staining on the intact control side and were used to measure levels of olfactory nerve degeneration and regeneration within the olfactory bulb, as follows:

$$\%\text{olfactory nerve innervation in the glomerular layer}=\frac{\frac{G-X-\mathrm{gal}\;\text{distance of}\;\mathrm{NTx}\;\text{side}}{G-P\;\text{distance of}\;\mathrm{NTx}\;\text{side}}}{\frac{G-X-\mathrm{gal}\;\text{distance of control side}}{G-P\;\mathrm{gal}\;\text{distance of control side}}}\times 100\%$$

### Immunohistochemical assessment

Immunohistochemical staining for glial fibrillary acidic protein (GFAP) and cluster of differentiation 68 (CD68) glycoprotein was performed on horizontal sections at four different time points following left NTx injury, Day 5, 14, 42, and 100. GFAP is constitutively produced by astrocytes. In the reactive glial response to central nervous system injury, hypertrophic reactive astrocytes increase their expression of GFAP.^27^ CD68 staining was used to measure injury‐induced inflammatory changes at different time points after NTx injury. CD68 is a lysosomal membrane‐associated glycoprotein that is expressed on the surface of histiocytes, cells that are part of the immune system, including macrophages and microglia, and play an important role in phagocytic activities.

After washing with phosphate buffer saline (PBS) for 5 min, sections were processed by immersion for 1 min intervals in a series of alcohol solutions (70, 95, 100, 95, 70% ethanol). This was followed by three 5 min washes with 0.3% Triton X‐100 in PBS. Sections were then incubated with 5% normal goat serum, 1% bovine serum albumin, 0.5% Triton X‐100 in PBS for 30 min and reacted with one of the following primary antibodies: rabbit anti‐mouse GFAP antibody (1:500, DAKO, USA) and rat anti‐mouse CD68 antibody (1:100, AbD serotec, USA). These antibodies were visualized using Cy3‐conjugated goat anti‐rabbit IgG (1:100, GE, USA) and Alexa Fluor 488‐conjugated goat anti‐rat IgG (1:100, Invitrogen, USA) under fluorescent microscope, respectively. GFAP‐ and CD68‐positive cells were counted in five different 0.01 mm^2^ sampling areas located in the anterior (injured) region of the olfactory bulb (five areas: the anterior apex area, areas of anteromedial corner, and anterolateral corner of the olfactory bulb, and fixed midpoint areas between the anterior apex area and anteromedial and anterolateral corner areas). The average number of GFAP and CD68‐positive cells/0.01mm^2^ were then calculated for NTx mice at each of the four recovery time points.

### Olfactory function test

To determine if olfactory function recovered after the NTx, a smell detection test using avoidance conditioning behavior to cycloheximide was administered to mice before and after the NTx as reported previously.^13^, ^14^, ^15^ Cycloheximide has a peculiar odor and unpleasant taste for mice. Mice were first deprived of water for 48 h and then trained to avoid cycloheximide solution. Before NTx surgery, mice were conditioned in two or more training sessions, each consisting of 10 trials. In each trial, the mouse was presented with bottles of 0.01% cycloheximide solution and distilled water one positioned on the left the other on the right side of a test cage. When the mouse licked the delivery tube of either bottle, the bottles were withdrawn from view and presented again. The left and right positions of the two bottles were shifted according to the Gellermann series (cycloheximide bottle position: right (R)‐left (L)‐L‐R‐L‐L‐R‐R‐R‐L). Mice were considered to have learned the smell of cycloheximide when they chose the distilled water bottle 10 consecutive times out of 10 trials (percent score: 100%) on two consecutive test sessions. After NTx surgery, the test was administered every 7 days until the mouse regained its olfactory function (scored 10 out of 10 correct responses), or exceeded a 100‐day cutoff period. Mice that scored 100% at one of the recovery test days were considered to have fully recovered their olfactory function.

### Statistical analysis

All numerical data obtained are expressed as means ± standard error of the mean (SEM). For statistical analysis of the data, the Mann–Whitney *U* test was used to determine differences in average values between two groups. For three groups, the two‐way analysis of variance (ANOVA) was used and post hoc comparisons were performed by the Bonferroni's method. The chi‐square (*χ* ^2^) test for independence was used to test for differences in ratio. Differences were regarded as significant when *p* < 0.05 for two group and *p* < 0.0167 for three group comparisons.

## Results

### Effects of IgG injection

**Figure 2:**
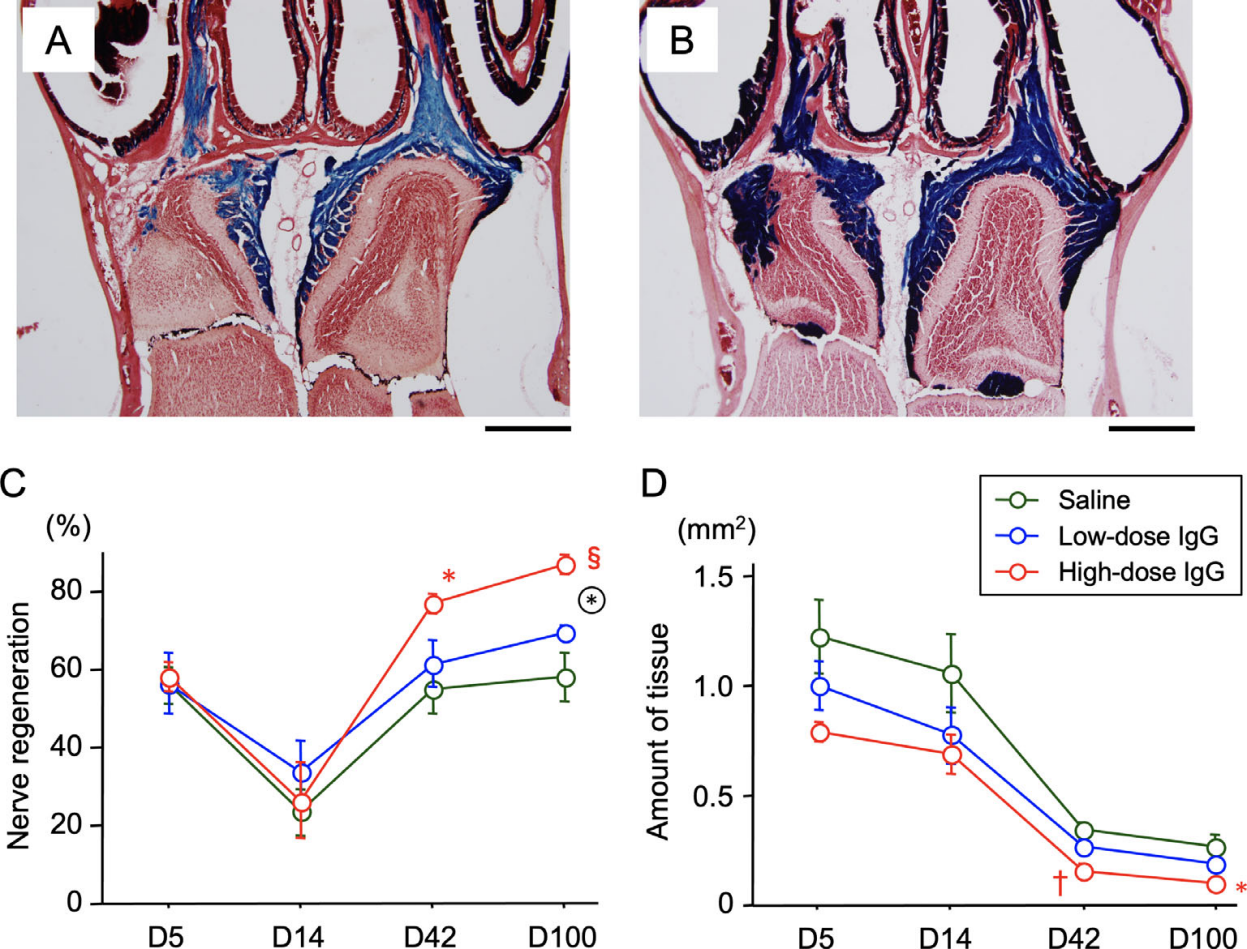
Effects of IgG on recovery from olfactory NTx injury. Histological sections illustrating control saline (Panel A) and high‐dose IgG (400 mg/kg BW, Panel B)‐injected mice at 100 days after NTx injury. Calibration bar = 500 μm. Quantitative measurements showing the time course and comparison of changes in the amount of injury‐associated tissue (C) and X‐gal stained olfactory nerve innervation to the glomerular layer on the olfactory bulb (D) for IgG (low‐ and high doses) and control animals. Significant differences are shown as * *p* < 0.0167, † *p* < 0.005, § *p* < 0.0005 compared to the control saline group. A circle surrounding an asterisk indicates a significant difference between the low‐ and high‐dose IgG groups (*p* < 0.0167).

To determine if high‐dose IgG treatment can facilitate recovery of the olfactory nerves after NTx injury, it was injected intraperitoneally in the severe injury model. Figure 2A shows results of the control (saline) compared to effects of IgG treatment (Fig. 2B) at 100 days after NTx injury. A decrease in the percentage of X‐gal (blue) staining on the NTx side at Day 5 and Day 14 reflect the degeneration of olfactory nerves **(**Fig. 2C). However, the subsequent increase in blue staining in the nerve and glomerular layers at Day 42 and 100 indicate that the regenerating olfactory nerves had reestablished connections with the olfactory bulb. Compared to the saline controls, a significantly higher level of the nerve recovery was found in the IgG‐injected mice at Day 100, and this increase was dose dependent.

Figure 2D shows changes in the amount of injury‐associated tissue (glial scar) present on the NTx side. The amounts increased at Day 5 and gradually decreased at Day 14 and after. The tissue amount in low‐ and high doses of IgG‐injected mice was significantly less than that in the control saline‐injected mice.

**Figure 3:**
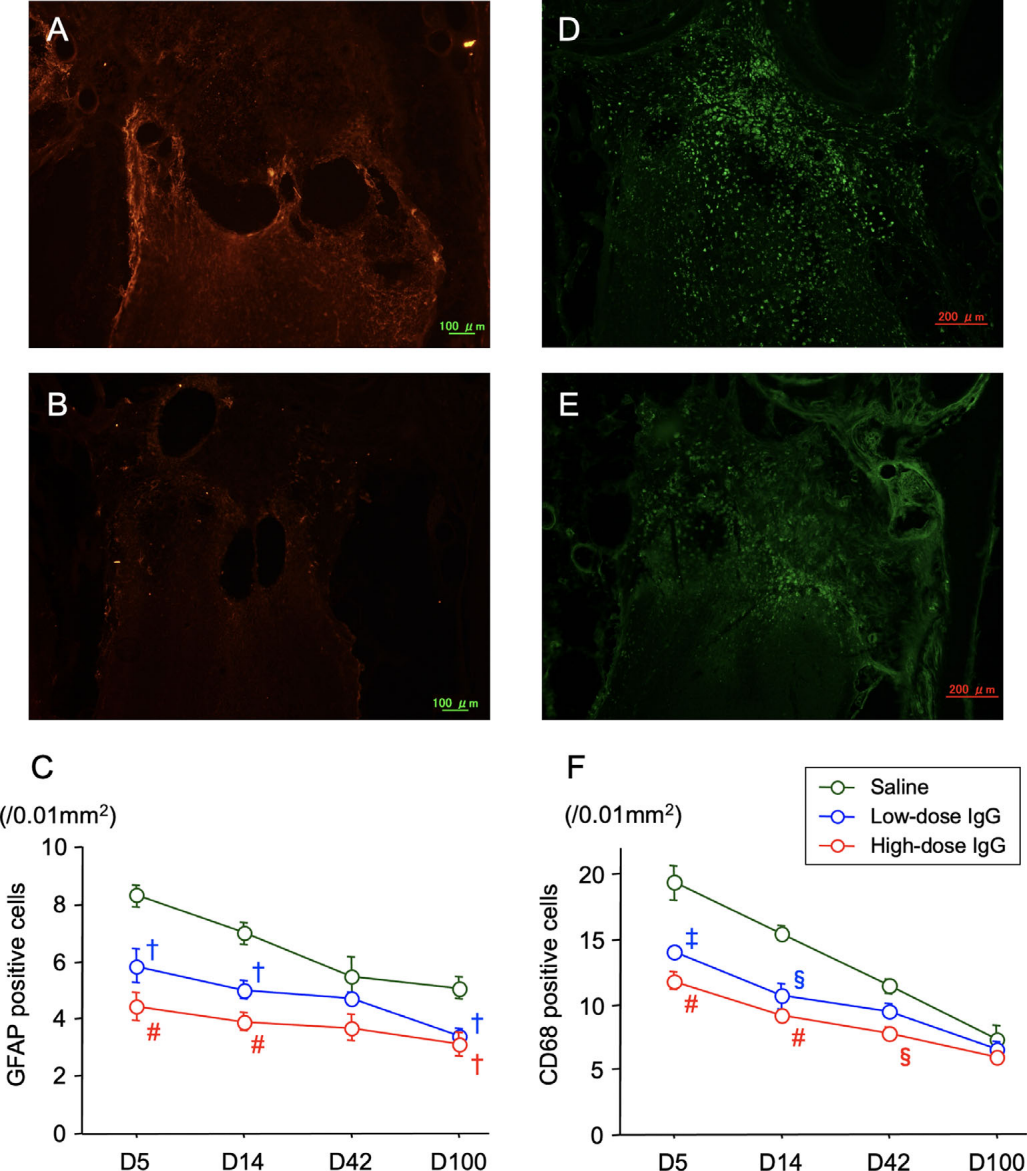
Effects of IgG on glial and inflammatory cells after NTx injury. Histological sections are from the left side (NTx side) of the head, left olfactory bulb for both GFAP (A and B) and CD68 (D and E) immunohistochemical staining at 5 days after the NTx injury. The upper panels show the saline controls (A and D) and lower panels the high‐dose IgG (400 mg/kg, B and E)‐injected animals. The two graphs plot quantitative measurements for the amount of GFAP‐positive cells **(C)** and CD68‐positive cells **(F)** for high‐ and low doses of IgG compared to saline controls. Data plotted are means ± SE. Significant differences are shown as † *p* < 0.005, ‡ *p* < 0.001, § *p* < 0.0005, # *p* < 0.0001 compared to the control saline group.

Both GFAP‐positive cells and CD68‐positive cells increased on the NTx side in the olfactory bulbs at Day 5 and gradually decreased at Day 14 and later recovery times (Fig. 3). With IgG treatment, the number of both GFAP and CD68 cells decreased compared with those in control mice in a dose‐dependent manner.

### Olfactory function tests

An olfactory function test using avoidance conditioning behavior to cycloheximide was administered to mice injected with high‐dose IgG (400 mg/kg) and control saline (35 ml/kg) before and after bilateral NTx. For the IgG group, eight of 13 (54%) mice achieved a score of 100% on the olfactory function test after NTx, indicating that their olfactory function had recovered. The average time required for behavioral recovery in the eight mice was 54 ± 9 days. For the control saline group, however, none of 14 (0%) mice recovered their olfactory function. High‐dose IgG‐injected mice showed a significantly higher rate of olfactory function recovery than control mice (*p* < 0.0006).

## Discussion

The present study shows that high‐dose IgG can suppress local infiltration of inflammatory cells and glial scar tissue formation and subsequently facilitate morphological and functional recovery from injury of the olfactory system. These results coincide with our previous reports that demonstrated olfactory nerve regeneration by systemic administration of steroids, anti‐IL‐6 receptor antibody, TNF‐α blocker, and anti‐HMGB1 antibody, all of which suppress local inflammation and glial scar formation in the mouse injury model.^12^, ^13^, ^14^, ^15^ Although IgG used in our study is different from those agents, the previous and present studies showed similar results, which suggests a possibility that any anti‐inflammatory treatment contributes to olfactory system recovery after nerve injury.

The mechanisms of IgG contribution to olfactory nerve regeneration can be explained as inactivation of inflammatory cells that produce pro‐inflammatory cytokines. The process of neural injury consists of two phases, the primary injury and the secondary injury.^28^ The primary injury is caused by direct mechanical damage and the injured neuronal tissue releases intracellular damage‐associated molecular patterns (DAMPs) as HMGB1 and heat shock protein, which activate macrophage/microglia via pro‐inflammatory membrane receptors such as Toll‐like receptors (TLRs) and the receptor for advanced glycation end products (RAGE).^29^, ^30^, ^31^ The activated cells mediate neuroinflammation by secreting nitrite and cytokines such as TNF‐α, IL‐1β, and IL‐6, thereby inducing necrosis of other neurons and resulting in further DAMP release. Thus, the secondary injury is a chemical damage exacerbated by a positive feedback circuit between DAMPs and pro‐inflammatory cytokines. These cytokines induce expansion of tissue damage by increase in vascular permeability, massive recruitment of inflammatory cells such as macrophages/microglia, neutrophils, and lymphocytes, which produce proteolytic enzymes and reactive oxygen species causing myelin degeneration and neuronal apoptosis, resulting in neurological deficits. IgG is composed of the Fcγ region and the F(ab’)_2_ region. Fcγ region can bind to Fcγ receptors expressed on the surface of these inflammatory cells, leading to inhibition of pro‐inflammatory cytokine release.^24^ Our previous studies revealed that local inflammatory reaction is reduced and restoration of injured olfactory nerve is facilitated both morphologically and functionally when activity of IL‐6 or TNF‐α is suppressed.^13^, ^14^ Therefore, efficacy of IgG to olfactory nerve regeneration can be reasonable.

Neuroinflammation is associated with reactive astrocytes since TLRs are expressed on astrocytes. Actually, GFAP expression was decreased by anti‐HMGB1 antibody.^32^ Therefore, once the primary injury occurs, DAMPs are inevitably released and activate astrocytes with an increase in GFAP expression, which induces gliosis and scar formation and enhance release of inflammatory cytokines, leading to limitation of axonal regeneration.^33^ Our previous study proved GFAP‐positive astrocytes to be a useful indicator of damage within the olfactory bulb since GFAP was significantly higher in severe injury models than that in mild injury for mice examined at different recovery periods.^12^ This suggests that a more prolonged residual tissue response may occur following severe injury. Astrocytes have been reported to express Fcγ receptor IIB, which is an inhibitory subtype of IgG receptors.^34^ Thus, IgG is considered to suppress astrocyte activation with GFAP expression via Fcγ receptor IIB and contribute to facilitation of olfactory nerve regeneration after injury.

One of the great advantages to use IgG for treatment is that its clinical safety is considerably promising. High‐dose IgG treatment has a history in which it has been introduced to many autoimmune and inflammatory diseases including neurological diseases associated with acute and chronic inflammation for several decades.^21^ There have been no or few clinical and experimental reports showing serious adverse effects due to in vivo administration of high‐dose IgG,^35^ compared with steroids, which are not recommended for patients with head injury because of no significant effects on morbidity and mortality and concerns about their adverse effects.^16^, ^17^, ^18^ Rather, high‐dose IgG has been shown neuroprotective efficacy against brain edema, ischemia, and subarachnoid hemorrhage in acute brain injury.^24^, ^31^ It is also useful for prevention of septic infection,^36^ while anti‐IL‐6 antibody and TNF‐α blocker sometimes aggravate infectious diseases because of oversuppression of the immune system.^19^, ^20^ Since high‐dose IgG is efficacious against other brain damages occurring together with olfactory dysfunction, it can be a preferable agent to use in severe head injury cases.

## Conflict of Interest

All authors declare no conflict of interest.

## Author Contributions

Conception and design of the study: M.K. Data acquisition and analysis: K.N., M.K., and E.I. Drafting the manuscript: M.K., K.N., and K.T. All authors reviewed and accepted the final draft of the manuscript. K.N. and M.K. contributed equally to this work.

## Ethics Approval

All protocols and surgical procedures for this study were reviewed and approved by the Institutional Animal Care and Use Committee of Mie University (No. 20–41).
